# Dual-Emission Origins in Bi^3+^-Doped *M*_2_O_3_ Sesquioxides (*M* = Sc, Y, Gd and Lu): A First-Principles Study

**DOI:** 10.3390/ma17092039

**Published:** 2024-04-26

**Authors:** Haonian Bai, Bibo Lou, Mekhrdod S. Kurboniyon, Andrzej Suchocki, Mikhail G. Brik, Jing Wang, Chonggeng Ma

**Affiliations:** 1 School of Science & Optoelectronic Engineering, Chongqing University of Posts and Telecommunications, Chongqing 400065, China; s210601001@stu.cqupt.edu.cn (H.B.); mehrdod-92@mail.ru (M.S.K.); mikhail.brik@ut.ee (M.G.B.); 2 Center of Innovative Development of Science and New Technologies, National Academy of Sciences of Tajikistan, Dushanbe 734025, Tajikistan; 3 Institute of Physics, Polish Academy of Sciences, Al Lotnikow 32-46, PL-02668 Warsaw, Poland; suchy@ifpan.edu.pl; 4 State Key Laboratory of Optoelectronic Materials and Technologies, School of Chemistry, Sun Yat-sen University, Guangzhou 510275, China; ceswj@mail.sysu.edu.cn

**Keywords:** bismuth, sesquioxide, dual emissions, Stokes shift, first-principles

## Abstract

Bi^3+^-doped sesquioxides exhibit dual emissions, marked by distinct Stokes shift and bandwidth, meaning unraveling their underlying origins is particularly intriguing. In this study, we employ first-principles calculations to investigate the luminescence mechanisms within the *M*_2_O_3_:Bi^3+^ (*M* = Sc, Y, Gd, Lu) series, with the goal of addressing the posed inquiry. Our investigation commences with the analysis of the site occupancy and charge state of bismuth ions in the two cationic sites through formation energy calculations. Additionally, we examine the local coordination environments for various excited states of Bi^3+^ dopants, including the ^3^P_0,1_ state and two types of charge transfer states, by evaluating their equilibrium geometric structures. The utilization of the hybrid functional enables us to obtain results of electronic structures and optical properties comparable with experiments. Importantly, the calculated energies for the 6s-6p transitions of Bi^3+^ dopants in the *M*_2_O_3_ series align well with the observed dual-emission energies. This alignment challenges the conventional spectroscopic sense that emission bands with large Stokes shifts can be exclusively ascribed to charge transfer transitions. Consequently, the integration of experimental and theoretical approaches emerges as the optimal strategy for designing novel Bi^3+^-doped phosphors.

## 1. Introduction

Due to the presence of the 6s^2^ lone pair, the photoluminescence of Bi^3+^ is sensitive to the ligand environment and ranges from ultraviolent to infrared in different matrices, making bismuth an excellent activator [1,2]. In particular, some single Bi^3+^-doped phosphors exhibit an extra red-shifted emission band in addition to the commonly observed green or blue emission [3,4,5]. This dual-mode emission characteristic is attractive for anti-counterfeiting, temperature sensing and healthcare lighting, e.g., the MgGa_2_O_4_:Bi^3+^ material exhibits dynamic luminescence evolution from near-infrared or green light to bluish-white light, showing excellent multi-mode dynamic anti-counterfeiting and encryption performances [6]. In order to improve the performance of the dual-mode emission in Bi^3+^-doped phosphors and to push their application boundaries, great efforts have been made to reveal the origin of emission bands and understand the luminescence mechanism. Several potential mechanisms have been proposed in different materials, including the site-selective occupancy of dopants [5,6], the influence of bismuth ions with lower valence states [7], the formation of Bi pairs or clusters [8] and the Jahn–Teller distortion of the local environment [9]. However, the spectra assignments of the Bi^3+^-doped phosphors were generally judged by the full-width at half maximum (FWHM) and the Stokes shift properties, where the emission bands with larger Stokes shift were always assigned to the charge transfer transitions in experiment. Obviously, such experiential spectra assignment methods were not explicit enough for understanding and developing dual-mode emission phosphors.

Bi^3+^-doped sesquioxides, such as the series of *M*_2_O_3_:Bi^3+^ (*M* = Sc, Y, Gd and Lu), are promising hosts that exhibit dual-mode emission. In *M*_2_O_3_ hosts, the *M*^3+^ cations have two different ligand sites, namely S_6_ and C_2_ sites, both of which are hexacoordinated, and the six O^2−^ ions form highly symmetric octahedral coordination in the S_6_ site [10]. Previous research has focused on elucidating the assignment of the narrow-band emission and the broad-band emission with larger Stokes shift to the two Bi^3+^ sites in the *M*_2_O_3_:Bi^3+^ series. It has been found that Bi^3+^ ions mainly occupy the central position of the highly symmetric octahedron, which contributes to the stable narrow-band emission with small Stokes shift, e.g., the emission of Lu_2_O_3_ and Gd_2_O_3_ with 35 and 40 nm FWHM, respectively, as reported by Z. Zhang [11]. However, the detailed geometric and electronic properties have hardly been studied to understand the remarkable difference in the Stokes shift of the two cation sites, and the possibility of the charge transfer transition has not been completely ruled out. First-principles calculations have been widely used as a powerful tool to investigate and elucidate the excitation, relaxation and emission processes of a large number of bismuth-doped phosphors [12]. Density functional theory (DFT) can effectively simulate the energy of the ground state and excited states of Bi ions with different valence states in activated phosphors, as well as the optical transitions of Bi^3+^ dopants in phosphors, including the ^1^S_0_ → ^3^P_0,1_ inner Bi^3+^, the charge transfer transitions including the valence band to Bi^3+^ charge transfer (CT) transitions or the Bi^3+^ to conduction band, i.e., the metal-to-metal charge transfer (MMCT) transition.

Here, the luminescence mechanism of the dual-mode emission of Bi^3+^ ions in *M*_2_O_3_ (*M* = Sc, Y, Gd and Lu) matrices was investigated using the first-principles methods. The site occupancy and the valence state of the bismuth ions were confirmed by formation energy studies. The geometric and electronic properties of the ground and excited states were calculated to obtain the excitation, relaxation and emission processes of the series of phosphors, which provides insight into the origin of the dual-mode emission. Furthermore, the variation in the (BiO_6_)^3−^ ligand environment from the ground states to the excited states was studied for the two cation sites to help explain the remarkable difference in Stokes shift between the two emission bands.

## 2. Methods

The first-principles calculations were performed utilizing the Vienna ab initio simulation package [13], employing the projector augmented-wave method in conjunction with density functional theory (DFT). The Perdew–Burke–Ernzerof (PBE) functional [14] was adopted in the geometric structure relaxation, along with the consideration of the spin-orbit coupling interaction for Bismuth dopants. Semicore electrons were explicitly treated with the recommended projector augment-wave (PAW) pseudopotentials [15], including 4s^2^4p^6^5s^2^4d^1^, 2s^2^2p^6^3d^1^4s^2^, 5p^6^5d^1^6s^2^, 5p^6^6s^2^5d^1^, 2s^2^2p^4^ and 5d^10^6s^2^6p^3^ for Y, Sc, Lu, Gd, O and Bi elements, respectively. The supercell, containing 80 atoms, was employed in modeling the defect-doped systems, and one *k* point *Γ* was used in sampling the Brillouin zone. The cutoff energy of the plane-wave basis was set to 520 eV, and the convergent criteria were $10^{-5}$ eV for electronic energy minimization and 0.02 eV/Å for Hellman–Feynman forces on each atom. Based on the relaxed equilibrium geometric structures, the standard PBE0 functionals [16] were utilized to obtain better electronic structure and photoluminescence properties.

The formation energy of a defect *X* in the charge state of *q* can be derived as follows [17]:

(1) $$E^{f}(X^{q},E_{F})=E_{tot}\left[X^{q}\right]-E_{tot}\left[bulk\right]-\sum_{i}n_{i}\mu_{i}+qE_{F}$$

where $E_{tot}$ is the total energy of the optimized supercells, $n_{i}\;$is the number of atoms in elements *i*, which are added to ($n_{i}$ > 0) and or removed from ($n_{i}$ < 0) the perfect supercell, and $\mu_{i}$ corresponds to chemical potentials of these species. The Fermi energy level${\;E}_{F}$ represents the chemical potential of the electrons in the host. The thermodynamic charge transition level $\epsilon (q_{1}/q_{2})$ was utilized to predict the positions of defect levels. It is defined as the Fermi level at which the defect formation energies of $X^{q_{1}}$ and $X^{q_{2}}$ equal each other. It can be deduced from Equation (1) as:

(2) $$\epsilon (q_{1}/q_{2})=\frac{E^{f}\left(X^{q_{1}},\;{\;E}_{f}=0\right)-E^{f}\left(X^{q_{2}},\;{\;E}_{f}=0\right)}{q_{2}-q_{1}}$$

where post hoc corrections to the total energy of the charged defects are employed following the method proposed in Ref. [18].

For Bi^3+^ ion-doped system, the dominant emission can originate from the equilibrium structure of the 6s^1^6p^1^ (^3^P_0,1_) excited state and the charge transfer state, e.g., Bi^4+^+*e* and Bi^2+^+*h*, where the Bi^4+^+*e* is the excited state of charge transfer from Bi^3+^ ions to the bottom of the condition band and always denoted as MMCT, whereas the Bi^2+^+*h* is the excited state of charge transfer from the top of the valance band to Bi^3+^ dopants and always denoted as CT. As in our previous work [8], the equilibrium geometric structure of the MMCT excited state was approximated as $M_{2}O_{3}:{Bi}^{4+}$ by the geometric relaxation of the supercell that one cation was substituted by Bi^4+^ ions. Similarly, the CT excited state was approximated as $M_{2}O_{3}:Bi^{2+}$. The ^3^P_0,1_ excited state was approximated by constraining the electron occupancy to (6s_1/2_)^1^(6p_1/2_)^1^ for $M_{2}O_{3}:Bi^{3+}$, where the *6*s_1/2_ and *6*p_1/2_ are Kohn–Sham orbitals obtained with PBE+ SOC functional. Based on the relaxed equilibrium geometric structures, the peak energy of excitation or emission for a given transition can be obtained approximately by the differences of the total energies of the excited and ground electronic state following the Franck–Condon principle.

## 3. Results and Discussion

### 3.1. Properties of the M_2_O_3_ Hosts

**Figure 1:**
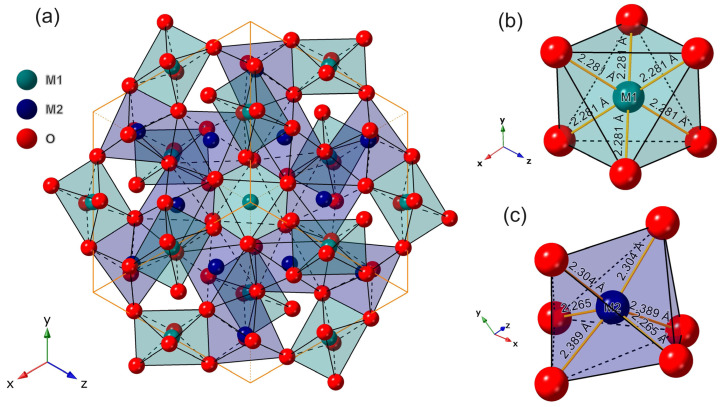
The crystal schematic diagram of the (**a**) *M*_2_O_3_ (*M* = Sc, Y, Gd and Lu) and the (**b**) S_6_ and (**c**) C_2_ cation crystallographic sites, where the bond lengths of Gd_2_O_3_ material were listed as example.

**Figure 2:**
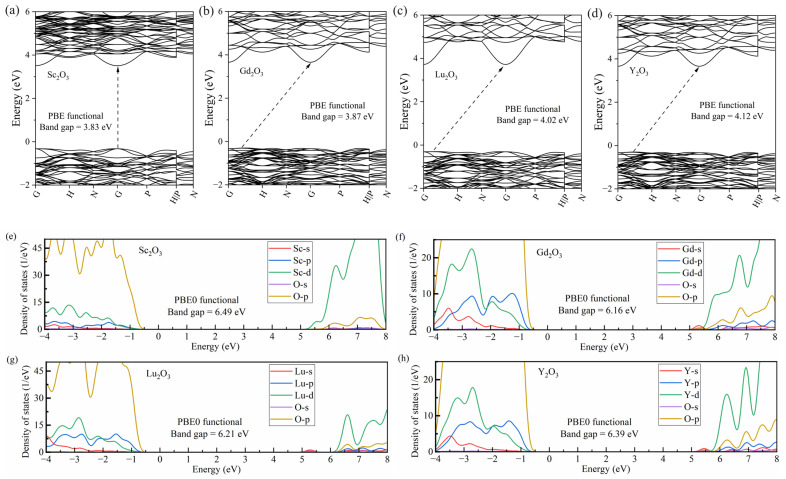
The PBE calculated band structure (**a**–**d**) and the PBE0 calculated DOSs (**e**–**h**) of a series of *M*_2_O_3_ (*M* = Sc, Gd, Lu and Y), respectively.

The *M*_2_O_3_ (*M* = Sc, Y, Gd and Lu) matrices are attributed to the cubic crystal system and *Ia-3* (No. 206) space group. There are two different cation crystallographic sites, C_2_ and S_6_, which can be replaced with Bi^3+^ ions, as shown in Figure 1. The crystal structure of cubic *M*_2_O_3_ is composed of regular and asymmetric octahedra of *M* and O atoms, with *M* atoms filling the interstitial sites of the octahedra. The band structures of four pristine *M*_2_O_3_ are plotted in Figure 2.

The results show that Sc_2_O_3_ is a direct band gap material, with the conduction band minimum (CBM) and valance band maximum (VBM) located at the high-symmetry *k*-point of *Γ*. For the remaining three hosts, Y_2_O_3_, Gd_2_O_3_ and Lu_2_O_3_, their VBM was located at the *k*-path from the high-symmetry *k*-point of *Γ* to *H*, implying indirect gap semiconductors. The Kohn–Sham band gaps calculated with the PBE functional are 3.83 eV, 3.87 eV, 4.02 eV and 4.12 eV for *M* = Sc, Gd, Lu and Y, respectively, which are seriously underestimated compared to the experimentally reported optical band gaps. Since the excitation and emission processes of the CT and MMCT states involve the orbitals of the band edges, the accuracy of the band gap values can greatly influence the spectral assignments and mechanism studies. Hybrid DFT has been widely used to improve the description of electronic structures. With the standard PBE0 functional, the band gap energy (*E*_g_) was improved to be 6.49 eV, 6.16 eV, 6.21 eV and 6.39 eV for *M* = Sc, Gd, Lu and Y, respectively. The PBE0 calculated band gap values are consistent with those estimated in the vacuum-referred binding energy diagram, reported by P. Dorenbos [19], which are about 6.50 and 6.40 eV for Sc_2_O_3_ and Y_2_O_3_, respectively. Furthermore, the choice of the PBE0 functional aligns well with the nonempirical hybrid functionals, characterized by a Fock exchange fraction inversely correlated with the material’s dielectric constant ($\epsilon_{\infty }$) [20], showing promise in achieving more uniform accuracy in band gap prediction, as well as in predicting other electronic and optical properties of semiconductors [21]. In Figure 2, the PBE0 calculated density of states (DOSs) exhibit the composition of the band edges. For all four hosts, the tops of the valence bands are dominated by the O-p orbitals, partially contributed by the orbitals of cations. For Sc_2_O_3_ hosts, the bottom of the conduction band was dominated by the Sc-d orbital. However, the bottom of the conduction band of the other three hosts was dominated by the *M*-s orbitals and partially mixed with *M*-p, *M*-d and O-p orbitals.

### 3.2. The Defect Properties of M_2_O_3_:Bi^3+^

**Figure 3:**
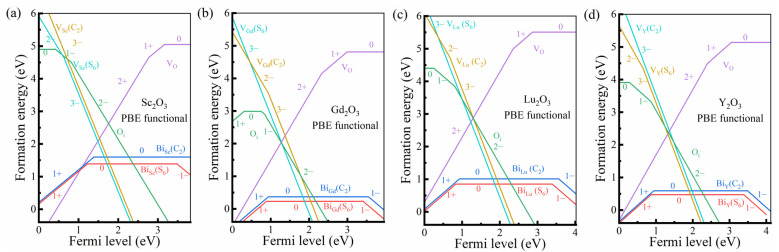
The PBE calculated formation energies of intrinsic defects and Bi^3+^ dopants in *M*_2_O_3_ sesquioxides (*M* = Sc (**a**), Gd (**b**), Lu (**c**) and Y (**d**)) as a function of the Fermi level in O-rich environment. It should be noted that the formation energy lines of Bi^3+^ dopants will be slightly shifted according to the actual doping concentration.

As shown in Figure 3, the formation energy calculations were performed for studying the properties of the intrinsic and Bi-doped defects in *M*_2_O_3_ (*M* = Sc, Y, Gd and Lu), including the intrinsic defects of cation vacancies at the C_2_ and S_6_ sites, e.g., *V_M_*(C_2_) and *V_M_*(S_6_), oxygen vacancies (*V*_O_) and oxygen interstitial defects (O_i_), as well as those of bismuth ions substituting the C_2_ and S_6_ cation sites.

By considering the synthesis conditions, the referenced chemical potentials of the O, *M* and Bi elements were set as follows:

(3) $$\mu_{O}=1/2E_{O_{2}\left[gas\right]}+\Delta \mu_{O}$$

(4) $$\mu_{M}=1/2\left(E_{M_{2}O_{3}\left[bulk\right]}-{3\mu }_{O}\right),$$

(5) $$\mu_{Bi}={1/2(E}_{{Bi}_{2}O_{3}[bulk]}-3\mu_{O}),$$

where $E_{M_{2}O_{3}\left[bulk\right]}$ and $E_{{Bi}_{2}O_{3}\left[bulk\right]}$ are the calculated total energy per formula unit for *M*_2_O_3_ and Bi_2_O_3_, respectively, and$\;E_{O_{2}\left[gas\right]}$ is the room temperature and partial-pressure-corrected chemical potential of oxygen gas. In our calculation, $\Delta \mu_{O}$ was set as 0 eV to simulate the oxygen-rich environment [22,23].

By studying the formation energies, the site occupancy and charge state of the intrinsic defects and bismuth dopants can be well determined. According to the formation energy diagrams, the electric neutrality of the four *M*_2_O_3_ (*M* = Sc, Gd, Lu and Y) hosts is maintained by the charge balance between the cation vacancies $V_{M}^{3-}$ and anion vacancies $V_{O}^{2+}$. The energy positions of the Fermi levels were obtained to be around the middle of the band gap but close to the VBM, which is consistent with the O-rich environment in our calculations. Compared with the intrinsic defects, including V_O_, V*_M_*(S_6_), V*_M_*(C_2_) and O_i_ defects, whose formation energies are all around 2.0 eV in the *M*_2_O_3_ series, the bismuth dopants show remarkably lower formation energies. The bismuth dopants are expected to be the dominant defects in *M*_2_O_3_:Bi^3+^ phosphors. It is noted that the doped bismuth ions are mainly in the trivalent charge state; thus, the energy positions of the Fermi levels are hardly influenced by the doping concentration of Bi^3+^ ions. Furthermore, Figure 3 shows that the formation energies of Bi^3+^ dopants in the cation sites with S_6_ symmetry are slightly lower (−0.1~−0.2 eV) than those with C_2_ symmetry. This is similar to the formation energy properties of the two cation vacancies, V*_M_*(S_6_) and V*_M_*(C_2_).

**Figure 4:**
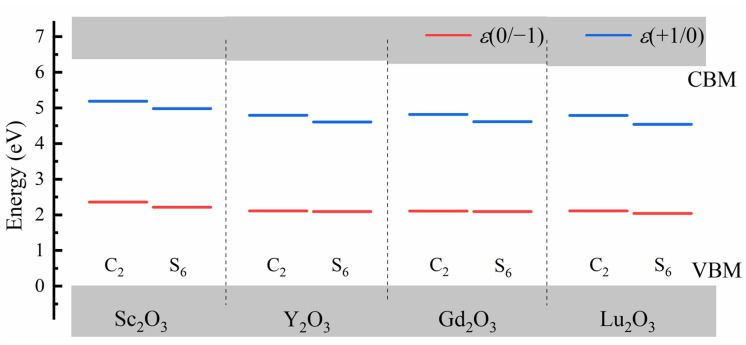
The thermodynamic charge transition levels of Bi^3+^ dopants, including *ε*(+1/0) and *ε*(0/−1) levels for Bi^3+^ ions in the two cation sites of *M*_2_O_3_ (*M* = Sc, Y, Gd and Lu), where the VBM is set as reference.

In Figure 4, the thermodynamic charge transition levels *ε*(+1/0) and *ε*(0/−1) of Bi dopants were plotted to investigate the trap properties of *M*_2_O_3_:Bi^3+^ in the four *M*_2_O_3_ states (*M* = Sc, Y, Gd and Lu).

The Bi^3+^ dopants can act as both the electron and hole trap in the four *M*_2_O_3_ hosts. The hole trap positions provided by the Bi^3+^ dopants in the C_2_ sites are 2.36 eV, 2.11 eV, 2.10 eV and 2.11 eV for *M* = Sc, Y, Gd and Lu, respectively. For the S_6_ cation sites with higher symmetry, the corresponding hole trap positions are 2.21 eV, 2.09 eV, 2.09 eV and 2.04 eV for *M* = Sc, Y, Gd and Lu referring to the VBM, respectively. The electron trap positions provided by the Bi^3+^ dopants are 1.24 eV, 1.60 eV, 1.45 eV and 1.42 eV at the C_2_ site for *M* = Sc, Y, Gd and Lu, respectively, while they are 1.45 eV, 1.78 eV, 1.65 eV and 1.67 eV at the S_6_ site for *M* = Sc, Y, Gd and Lu, respectively. For all four *M*_2_O_3_ hosts, the thermodynamic charge transition levels of Bi^3+^ dopants at S_6_ cation sites are slightly lower than those in the C_2_ cation sites; however, the energy difference between *ε*(+1/0) and *ε*(0/−1) levels is similar for Bi^3+^ in the two cation sites of sesquioxide and is hardly influenced by the local symmetry. Our calculation results are in great agreement with the vacuum-referred binding energy diagram provided by P. Dorenbos, where the MMCT transition energies are used to locate the Bi^3+^ 6s ground state relative to the CBM. It is reported that the Bi^3+^ dopants provide the hole trap and electron trap with depths around 2.0 eV and 1.0 eV, respectively, in both Y_2_O_3_:Bi^3+^ and Sc_2_O_3_:Bi^3+^.

**Figure 5:**
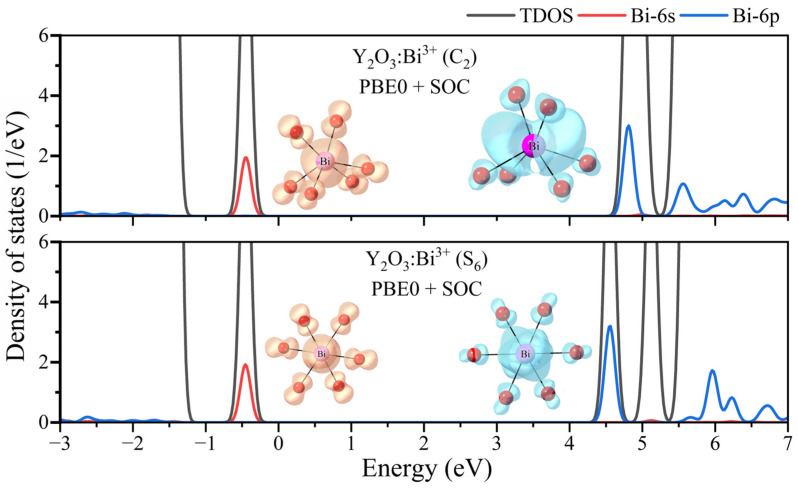
The PBE0+SOC calculated partial DOSs of 6s and 6p orbitals of Bi^3+^ dopants in the two cation sites for the ground state of Y_2_O_3_:Bi^3+^, and the corresponding charge density distribution profiles.

In order to investigate the electronic structure properties of Bi^3+^ dopants at the two cation sites, the PBE0+SOC calculated partial DOSs and the charge density distribution of 6s and 6p orbitals are plotted in Figure 5.

As the selected series of materials have similar structural environments and similar luminescent properties, only the DOSs of Y_2_O_3_:Bi^3+^ phosphors were provided. For the *M*_2_O_3_:Bi^3+^ series, the 6s and 6p orbitals of Bi^3+^ dopants are always in the band gap, regardless of Bi^3+^ substituting the S_6_ or C_2_ cation sites. Due to the stronger crystal filed environment at the S_6_ lattice site, the Bi^3+^ 6p orbital shows larger energy splitting than that at the C_2_ lattice site. As shown in the charge density profile, the Bi^3+^-6s orbital contains substantial contributions from the p orbitals of the six oxygen ligands, and the corresponding distributions are similar for Bi^3+^-6s orbitals at both the C_2_ and S_6_ sites. The charge density distributions of Bi^3+^-6p orbitals are quite different. The Bi^3+^-6p orbitals mainly distribute along the one axis for the Bi^3+^ ions at C_2_ sites; however, the charge density distributions of Bi^3+^-6p orbitals remain similar with the Bi^3+^-6s orbitals at S_6_ sites, where the six oxygen ligands contribute uniformly to the Bi^3+^-6p orbitals due to the high symmetry. The geometric structure relaxation and the Stokes shift properties of Bi dopants are correlated with the electronic structure of the Bi 6s and 6p orbitals, which results in the different luminescence properties of the ^3^P_0,1_ excited state at the two cation sites.

### 3.3. The Luminescence Mechanism of M_2_O_3_:Bi^3+^

As reported in experimental research [5.10], Bi^3+^ dopants in a series of *M*_2_O_3_ (*M* = Sc, Y, Gd, Lu) phosphors show similar excitation and emission characteristics, including a narrow-band absorption peak at 340–400 nm, corresponding to a strong emission in the 380–470 nm range, and a second localized band absorption in the 300–360 nm range, corresponding to a broad emission in the 360–600 nm range with low emission intensity. Table 1 shows the optical transition energies of Bi^3+^ ions in the four hosts, including the results from DFT calculations and the experimental reports, where the calculated excitation and emission energies are in good agreement with the experimental data [10].

For *M*_2_O_3_:Bi^3+^ phosphors, the *A* band transition dominates both the excitation and emission, and the CT and MMCT transitions require remarkably higher excitation energies. Our results support the *A* band assignment in previous research [5,10], where the broad excitation and emission bands were attributed to the Bi^3+^ dopants at C_2_ sites, and the sharp excitation and emission bands originated from the S_6_ sites. Except for the dominant *A* band optical transitions, G. Blasse reported a high-energy excitation band at 4.71 eV for Y_2_O_3_:Bi^3+^ [24], and Bordun observed similar excitation bands in Bi^3+^-doped Y_2_O_3_ and Sc_2_O_3_ ceramics [25]. R. K. Datta also reported that these are practically identical in both spectra, namely, a weak broad line around 4.70 eV and a slowly rising structure starting at 5.20 eV⁠ in Y_2_O_3_:Bi^3+^ [26]. Although prior studies assigned these bands to the *C*-band, P. Dorenbos agreed with the assignment of P. Boutinaud, who both suggested that these excitation bands should be assigned to the MMCT transition [27], and this is confirmed by our calculation results.

**Figure 6:**
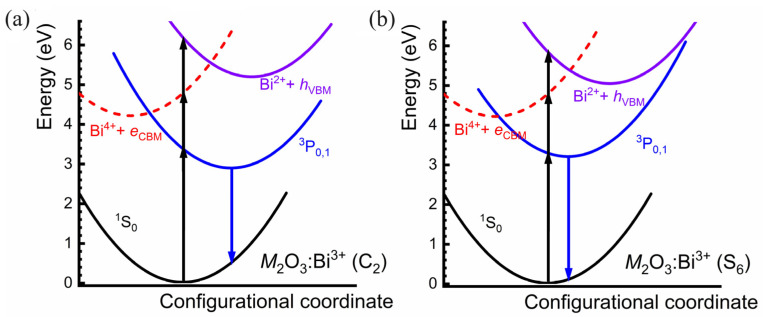
The configuration coordinate diagrams of the potential surfaces of Bi^3+^ dopants substituting the cation sites with (**a**) C_2_ symmetry and (**b**) S_6_ symmetry for *M*_2_O_3_:Bi^3+^ phosphors (*M* = Sc, Y, Gd and Lu), where ^1^S_0_ and ^3^P_0,1_ denote the ground and the lowest triplet 6s6p excited states of Bi^3+^, respectively, Bi^4+^ + *e*_CBM_ simulates Bi^4+^ with one electron at CBM, representing the lowest MMCT excited state, and Bi^2+^ + *h*_VBM_ simulates Bi^2+^ with a loose hole at VBM, representing the lowest CT excited state.

As listed in Table 1, there are slight differences (~0.3 eV) between the excitation energies of Bi^3+^ dopants being at the C_2_ and S_6_ sites, including the *A* band, CT and MMCT transitions. However, the emission properties of the A band transition are quite different for Bi^3+^ dopants at the two cation sites. In both of the experimental and calculation results, the Bi^3+^ dopants at C_2_ sites exhibit a large Stokes shift in the range of 1.2–1.4 eV, while such values are remarkably smaller (at around 0.3 eV) for the Bi^3+^ dopants at S_6_ sites. There were arguments that the main double-peaked green emission band centered around 3.7 eV should correspond to the crystal-field splitting of the low-excited levels in the low-symmetry C_2_ luminescent center [28]. However, the energy positions of the lowest Bi^3+^ 6p levels show slight differences between the two sites, as plotted in Figure 5. And the geometric relaxation difference in the ^3^P_0,1_ excited state results in a remarkably different Stokes shift at the two sites according to our calculations. Based on the relaxed equilibrium structures of the ground state and various excited states of Y_2_O_3_:Bi^3+^, a schematic configuration coordinate diagram (Figure 6) is constructed to show all the excited states, illustrating the luminescence process. For the *M*_2_O_3_:Bi^3+^ series, the potential energy surface of the Bi^4+^ + *e*_CBM_ state intersects with that of (Bi^3+^) ^3^P_0,1_, and non-radiative relaxation from the former to the latter can be bridged with the cooperation of phonons.

This relaxation also occurs from Bi^2+^ + *h*_VBM_ to Bi^4+^ + *e*_CBM_ and (Bi^3+^) ^3^P_0,1_. The dominant emission is expected to originate from the lowest ^3^P_0,1_ excited state, and the Bi^3+^ dopants at C_2_ site exhibit obviously larger geometric relaxation in emission processes. The Stokes shift properties of Bi^3+^-doped phosphors are always considered as an important index to distinguish the emission band from the ^3^P_0,1_ and charge transfer states, e.g., the broad yellow light of Ba_2_YGaO_5_:Bi^3+^ peaking at 587 nm with full-width at half maximum (FWHM) of 135 nm was assigned as the MMCT transition due to the large Stokes shift (1.43 eV) [29]. Our calculations on the *M*_2_O_3_:Bi^3+^ series imply that the A band transition can also provide emission with large Stokes shift, and the detailed structure–activity relationship requires further studies.

## 4. Conclusions

In this study, we employed first-principles calculations to elucidate the luminescence mechanisms underlying the dual emissions in the *M*_2_O_3_:Bi^3+^ series (*M* = Sc, Y, Gd, Lu). Utilizing formation energy calculations, we confirmed the prevalence of trivalent bismuth ions in both cationic sites, with a slight preference for the *M*^3+^ sites with *S*_6_ symmetry over the *C*_2_ sites. The larger band gap of the *M*_2_O_3_ series, ranging from 6.1 to 6.5 eV, provides enough space to accommodate both the 6s and the lowest 6p orbitals of the Bi^3+^ dopants. Our calculated charge transition levels for Bi^3+^ dopants exhibit remarkable similarity in the two cationic sites of the *M*_2_O_3_ series, where Bi^3+^ ions can serve as deep electron traps (with depths around 1.0 eV) and hole traps (with depths around 2.0 eV). The dominance of the 6s-6p transitions of Bi^3+^ dopants in both cationic sites was affirmed, while the observed high-energy excitation band at approximately 4.7 eV was identified as the MMCT transition. Through an exploration of the variations in electronic and geometric structures during the luminescence kinetics processes, our calculations contribute to a comprehensive understanding and provide design principles for novel Bi^3+^-doped phosphors.

## Figures and Tables

**Table 1:** The calculated and measured excitation and emission energies of *M*_2_O_3_:Bi^3+^ series.

	MMCT		CT		*A* Band		Spectra Data ^1^	
	Exc.	Emi.	Exc.	Emi.	Exc.	Emi.	Exc.	Emi.
Sc_2_O_3_ (C_2_)	4.70	3.53	6.10	4.31	3.57	2.33	3.70	2.46
Sc_2_O_3_ (S_6_)	4.88	3.56	5.71	4.39	3.35	3.08	3.35	3.05
Y_2_O_3_ (C_2_)	4.97	3.84	5.85	3.72	3.62	2.29	3.70	2.55
Y_2_O_3_ (S_6_)	5.11	3.90	5.54	3.96	3.29	3.00	3.35	3.02
Gd_2_O_3_ (C_2_)	4.84	3.65	5.84	3.76	3.59	2.30	3.70	2.48
Gd_2_O_3_ (S_6_)	4.96	3.74	5.51	3.97	3.33	3.00	3.29	2.96
Lu_2_O_3_ (C_2_)	4.88	3.71	5.93	3.82	3.77	2.31	3.78	2.63
Lu_2_O_3_ (S_6_)	5.11	3.90	5.58	3.97	3.47	3.09	3.34	3.05

^1^ Refs. [10,11].

## Data Availability

Data are contained within the article.
